# Natural environments, ancestral diets, and microbial ecology: is there a modern “paleo-deficit disorder”? Part I

**DOI:** 10.1186/s40101-015-0041-y

**Published:** 2015-01-31

**Authors:** Alan C Logan, Martin A Katzman, Vicent Balanzá-Martínez

**Affiliations:** CAMNR, 23679 Calabasas Road Suite 542, Calabasas, CA 91302 USA; START Clinic for Mood and Anxiety Disorders, 32 Park Road, Toronto, ON M4W 2 N4 Canada; Department of Medicine, Section of Psychiatry and Psychological Medicine, University of Valencia Medical School, Avda. Blasco Ibáñez, 15, E46010 Valencia, Spain

## Abstract

Famed microbiologist René J. Dubos (1901–1982) was an early pioneer in the developmental origins of health and disease (DOHaD) construct. In the 1960s, he conducted groundbreaking experimental research concerning the ways in which early-life experience with nutrition, microbiota, stress, and other environmental variables could influence later-life health outcomes. He also wrote extensively on potential health consequences of a progressive loss of contact with natural environments (now referred to as green or blue space), arguing that Paleolithic experiences have created needs, particularly in the mental realm, that might not be met in the context of rapid global urbanization. He posited that humans would certainly adapt to modern urban landscapes and high technology, but there might be a toll to be paid in the form of higher psychological distress (symptoms of anxiety and depression) and diminished quality of life. In particular, there might be an erosion of humanness, exemplified by declines in altruism/empathy. Here in the first of a two-part review, we examine contemporary research related to natural environments and question to what extent Dubos might have been correct in some of his 50-year-old assertions.

“*Human beings can almost certainly survive and multiply in the polluted cage of technological civilization, but we may sacrifice much of our humanness in adapting to such conditions…The maintenance of biological and mental health requires that technological societies provide in some form the biological freedom enjoyed by our Paleolithic ancestors*”.

Dr René Dubos, Invited Editorial, *Life* Magazine, 1970 [1].

## Introduction

Global urbanization is expected to continue with rapid pace over the next several decades, with an additional 1.35 billion people expected to take up residence in cities within the next 15 years [2,3]. Properly planned and managed urbanization, along with the technologically assisted transition from developing to developed areas/nations, has the potential to promote a host of societal benefits. These include, but are certainly not limited to, undoing poverty, improving sanitation, reducing resource consumption, enhancing efficient commerce and trade, providing educational opportunities, easing of communications, as well as the efficient delivery of government and health-care services. However, translating the promissory notes of urbanicity into realized improvements in future city and metropolitan quality of life is not a simple task [4,5].

The health problems associated with rapid urbanization are profound, most notably the chronic non-communicable diseases (NCDs)—e.g., mental health disorders, and obesity and its correlates of type II diabetes, metabolic syndrome, and cardiovascular disease [6-13]. Urbanization, particularly in deprived areas, may drive changes in behavior that contribute to NCD risk—low physical activity, compromised sleep, and unhealthy dietary choices [14-17]. The progressive movement away from less sanitized traditional lifestyles has also altered the diversity of microbial contact [18]. Much has been written regarding environmental variables, including Westernized dietary patterns and increasing microbial sanitization, and their association with marked increases in allergic and autoimmune conditions in developed nations—as much as tenfold higher vs. developing nations [19-21]. The relationship between these variables and mental health is now an emerging area of research [22,23].

Some 4 decades prior to these and other chronic medical conditions being described as “epidemic” and “crisis level,” at least one scientist—René J. Dubos (1901–1982)—was providing early warning of impending shifts as they relate to urbanization, loss of biodiversity, and purported technological advances that were in reality (according to Dubos) unrelated to the promotion of human quality of life. Dubos gained his initial fame as a bench microbiologist, the discoverer of the first clinically tested antibiotic (gramicidin, 1939) [24]; over time he would become a noted expert in the field of early environmental influences in long-term health [25] (now referred to as developmental origins of health and disease (DOHaD)) and a Pulitzer-Prize-winning author (*So Human an Animal*, 1969). A prolific writer, Dubos left a trail of the literature on a wide variety of topics—much of which included a remarkable artistic synthesis of the medical sciences, humanities, and natural and social sciences.

As a highly respected scientist, Dubos understood the value of the most reductionist techniques in the process of scientific discovery; only through detailed and labor-intensive examination of isolated mechanisms was he able to uncover the ability of select soil microbes to combat disease-causing bacteria. However, he could also recognize the broad relevance of isolated findings to ecological systems, discussing interrelatedness and connectivity of anthropological and biopsychosocial variables at so many turns. These attributes helped to provide a unique ability in sewing together—both for the scientist and the lay reader—what would seem to be, at least at first glance, distinct and unrelated lines of scientific inquiry. He demonstrated the large-scale saliency of formerly compartmentalized research with an emphasis on personal, societal, and planetary health—all within the context of a rapidly changing, technologically focused world.

Here in this review, the authors will examine, with the benefit of contemporary research findings, to what extent Dubos appears correct in some of his assertions. More specifically, our focus will center on his contention that modern disconnects from ancestral influences—natural environments, traditional dietary practices, and incidental exposure to non-pathogenic microbes—would make itself known in health and well-being statistics (or outcomes related to humanness such as empathy). Dubos, as evidenced by his own words quoted throughout this review, underscored the importance of these variables. Moreover, from the ecological standpoint of health—both personal and planetary—he expressed that there was essentially no distinction between clear-cutting Giant Sequoia forests and doing the same to the non-harmful microbes that reside in the human gut. For Dubos, both practices had untold consequences. He argued that discussions of natural environments, mental health, and the principles of conservation were one in the same.

To be clear at the outset, Dubos was not anti-urban. A city-dweller himself, he wrote often on the many benefits of urban life and its ability to provide a rich milieu for mental growth. He understood that complexities of communities, and the socioeconomic status of those who reside within them, ensure that health does not exist on a neatly defined urban–rural divide. He was a scientist who embraced progress and the highest standards of scientific methodology; however, he was a staunch critic of technological expansion that is simply dressed up as “progress”, or more specifically, those developments in which mere convenience or entertainment potential is cloaked under the guise of improving quality of life.

Decades before “paleo” would become a trendy health topic, a niche “lifestyle,” or a way of dining that might involve the consumption of relatively expensive food choices [26], Dubos wrote extensively about the health implications of ignoring the indelible Paleolithic inscriptions within the genetic profile of the modern human. Although he wrote on a wide variety of topics, the evolutionary vs. contemporary environmental mismatch was a central theme. The disconnection from nature and ultra-rapid distancing from the environmental influences that shaped us over the millennia, he warned, could easily manifest in chronic disease and compromised mental health.

Dubos argued that because humans are very adaptable, the relationship between an evolutionary mismatch and erosion of health would be stealth-like; there would be only minimal awareness of the association, especially early on in the era of high technology and urbanization. In other words, it would be difficult for the general population and even health-care providers to make connections between current ill health and exceeding adaptive limits in the years or decades prior. Moreover, since humans are also attracted to gadgets and, as Dubos argued, “*seem to accept willingly, and indeed to enjoy*” many of the biological stresses of mega-city life, it would be even more difficult to appreciate ancestral needs that might be *missed* in the modern environment [27].

According to Dubos, humans, as a species, would make the necessary adaptations for *survival* in the technological and increasingly urbanized environment. However, survival could not be equated to optimal health or quality of life, and such adaptations may not be without cost to the individual and ultimately to society. The detrimental health effects of the slow and insidious presence of physical and psychological toxins, disturbed circadian rhythms, artificial foodstuffs (all acting in concert with the absence of nature interaction), would bypass identification by most individuals—rather, according to Dubos, they would only be apparent in the overall health statistics of the larger nature-disconnected population over time.

Beyond the specific health effects, he also hypothesized a corrosion of our “humanness,” where altruism and empathy would be particularly sensitive to deterioration if ancestral environmental necessities were diluted. In his words:

“*Man seems to be adapting to the ugliness of smoky skies, polluted streams, and anonymous buildings; to life without the fragrance of flowers, the song of birds, and other pleasurable stimuli from nature. This adaptation, however, is only superficial, and destructive in the long run. Air, water, earth, fire, the subtle forces of the cosmos, the natural rhythms and diversity of life have shaped man’s nature during the evolutionary past and have created deep-rooted sensual and emotional needs that cannot be eradicated. The impoverishment of sensual and emotional life will progressively result in the atrophy of our uniquely human attributes*” [28].

## Essential background

Before we examine the unfolding research within our three primary sections—natural environments, traditional diets, and microbiota—it seems critical to discuss the contextual framework in which this research resides. Discussions of natural environments (usually under the green space lexicon), traditional diets, and microbiota are often placed into contemporary research and policy silos. Not only are they separated from one another, but they are rarely placed into larger context of what should be obvious, related discussions.

Changes in screen-based media consumption, with massive increases in the hours devoted to so-called screen time over the last several decades, provide a critical example of contextual framing [29,30]. Indeed, compared to 1960, the *per capita* time spent devoted to media-based information consumption has increased by 60% [30]. From an evolutionary perspective, the same dopamine systems that signal ancient rewards—like food and water—also signal for information-seeking. In other words, turning on a smartphone or checking e-mail presents an opportunity to seek rewarding information, and that behavior has the potential to be reinforced [31].

It is not our contention that increases in information consumption are without benefit to human health and well-being. However, given that each day has a fixed 24-h period, the shift toward info-consumption raises obvious questions. As humans spend more time with screen media, are there specific activities or behaviors that have since declined? And, if so, what might be *missed* from a health perspective? If only through displacement, what might humans no longer be doing, or doing significantly less of, during this 24-h period? Less physical activity, less face-to-face social interactions, and/or less time spent in natural environments?

Screen media consumption is a cultural phenomenon with relevance to each of the main sections of our review—it does not stand alone. Research, admittedly only correlational, indicates that modern technological gadgets and the draw of screen-based media may be contributing to a displacement of nature-based recreation [32-34]. Over the last decade in North America, research indicates that adults and children are spending less time outdoors and significantly more time indoors [35]. The implications are obvious, and we will touch on these in more detail throughout our review—less contact with microbial diversity, changed dietary habits (which, in turn, alter intestinal microbial diversity), and less frequency of the Stone Age nature experiences Dubos considered to be necessary. Could there be a synergy between *excess* screen media consumption, high cognitive load, and *limited* time in nature? We will also add the variable of nutrition to this question later. Only through breaking down the silos might we understand if the detrimental health effects may be greater than the sum of these individually researched parts.

Excess screen-based media consumption, information load, and so-called techno-stress have recently been linked with poor psychological health [36-47]. Notwithstanding the potential benefits of media that may specifically foster helping behavior, overall daily screen media use is associated with diminished empathy and the ability to read facial emotions (and other non-verbal cues) that indicate the emotion felt by another [48,49]. In 1970, Stanley Milgram published his “urban overload” theory, which posited that the cognitive demands placed on an individual in complex urban environments would diminish the ability to recognize the social cues that might otherwise evoke empathy and altruism [50]. In its wake, a series of experimental studies showed that high levels of cognitive load from environmental inputs were associated with diminished helping behavior—ranging from granting a favor in an office setting, returning an experimental “lost” letter, saving recyclables for a fictitious art project, to helping an individual locate a lost contact lens [51].

However, Milgram could not have imagined the extent to which global, mobile communication technologies and internet access would factor into the cognitive load of contemporary environments [52]. The overwhelming amount of consumer choices in contemporary society only adds to the decision-making load [53]. Cognitive load has been shown to reduce the subjective experience of empathy and diminish neural activity in brain regions associated with empathy [54]. Interestingly, investigations involving adults from remote rural regions (living semi-nomadic lifestyles) have shown that, compared to urban residents, they are far less distracted by irrelevant information during cognitive tasks [55].

Evidence does show that urban (vs. rural) upbringing is associated with elevated cortisol responses to acute stress and a blunted cortisol awakening response [56]. Some research shows that attention-deficit hyperactivity levels are higher in urban areas, yet the reasons for an urban–rural gradient remain unclear [57,58]. At this point, it is unknown whether provider-diagnosed ADHD—which has increased threefold in children/teens in the US since 2003 and can vary by as much as 60% from county-to-county within the same US State [59,60]—is a surrogate marker of the pervasive distraction in urban environments.

More relevant to erosion of humanness, emerging research in experimental and real-world settings, shows that the mere presence of mobile communication devices may erode the quality of face-to-face interactions, diminishing closeness, connection, and empathic concern [61,62]. Dubos wrote on the topic in 1974:

“*Human crowds per se are not responsible for the nervousness of life in modern agglomerations. We suffer less from contact with people than from exposure to the unnatural stimuli generated by the machines that accompany them everywhere in the industrial world. Motor cars, motorcycles, telephones, radios, television sets, and other gadgets enslave us to a nonhuman and often antihuman environment*” [63], [page 104].

Prospective studies suggest that excess screen time may predict subsequent mental health problems. However, screen time research is complex. For example, research shows that in neighborhoods where walkability is less than optimal, screen time is higher [64,65]. Moreover, children residing in urban environments [66] and deprived neighborhoods [67] have higher daily screen time than rural or affluent counterparts. Screen time as it relates to diminished psychological well-being clearly interacts with physical activity, sleep quality, and social support [68,69], yet it may also be an independent variable in the risk of depression [70].

Research also shows that screen time (independently of physical activity) is significantly associated with the consumption of high-energy, low-nutrient foods and beverages [71]. Prospective research shows that baseline screen time predicts higher consumption of sugar-rich beverages when queried 20–24 months later [72,73]. Similar results have been reported concerning baseline screen time and subsequent dietary habits in high school students and young adults—more screen time predicted consumption of fast food, snacks, and high-energy foods and beverages 5 years later [74]. Indeed, total screen time, vs. sedentary time *per se*, is a better predictor of unhealthy dietary habits among children [75].

Also, crucial to the framing of our review are the massive strides which have been made in the area of evolutionary psychology and the ways in which the findings within the discipline interact with the broad aspects of environmental psychology research [76,77]; the fruits of this labor have made it clear that the brain regulates physiology and behavior via adaptive specializations established via a multi-generational “camping trip” spanning tens of thousands of years [77]. The generalized findings also force us to acknowledge that environmentally influenced aspects of cognition and behavior can occur outside of conscious awareness. Functionally organized systems within the brain were developed out of Stone Age problem-solving necessity, and they still weave their way through the factors with which contemporary mental health clinicians and scientists concern themselves—attention, reasoning, emotion, motivation, and learning [77].

While evolutionary and environmental psychology are expansive domains that have been the subject of recent expert reviews [76,77], in our current context of Dubos, some of the emerging research is worth specific discussion. For example, humans have been shown to rapidly and accurately detect even minor changes in the color or positioning of animals (vs. vehicles, tools, buildings, and other objects) within complex photographic scenes [78,79]. Moreover, when subjects are asked to process critical survival-based information in differing experimental contexts (ancestral grassland vs. modern city), there is improved memory in the ancestral setting [80]. Perceptual bias and differential physiological responses for the sights and sounds of ancestral threats (e.g., reptiles or spiders) are detectable even in infants, challenging the idea that social- and experience-based general learning can fully explain common human fears that likely have evolutionary roots [81,82].

The attentional privilege toward animals appears to be a product of a specialized visual recognition system shaped by ancestral selection pressures [79,83]. There is also evidence that subsequent to a visit to an urban farmer’s market, human spatial memory (the ability to recall select locations within a ≈ 90 vendor marketplace) is more accurate for locations where foods had a high caloric density. This finding was not related to food preferences or taste-driven desire for those foods, as this had been evaluated prior to the spatial memory exam and subsequently controlled. More likely, the accuracy reflects the ancestral foraging experiences where survival necessitated the securing of nutritionally dense plant foods [84]. Other researchers have found that human preference for the shininess of contemporary glossy (vs. matte) objects appears to be stem from millennia of sourcing a daily supply of fresh, flowing water [85].

While such background research does not necessarily substantiate Dubos’s argument that modern urbanites may experience an evolutionary mismatch between certain psychological requirements and the ability of the contemporary environment to help fulfill those needs, it does highlight that the Pleistocene era is still very much (functionally) alive in the brain of the modern human. In addition to the ancestral underpinnings, there are now volumes of research demonstrating the subtle, yet very meaningful, ways in which human cognition and emotion is influenced by environmental variables. The research findings in this area are not simply confined to the effects of noise, lighting, and colors [86-88], they run the gamut from sub-conscious fast-food logo exposure provoking impatience and mitigating the experience of pleasure [89,90], to the ability of subliminal ambient odors (at miniscule levels outside of olfactory awareness) to alter mental performances, preferences, and behavior [91-93]. Again, this background research does not validate the Dubos mismatch theory *per se*. However, the emerging research does support the broad notion that humans are environmentally influenced in a stealth-like manner, and that as Dubos stated, deterioration in the quality of the physical and social environment “*can degenerate without the persons involved being conscious of the loss this entails*” [94].

### Dubos on privacy

“*Freedom and privacy may come to constitute antisocial luxuries, and even to involve hardships. In consequence, the human beings most likely to prosper in congested urban environments will be those willing to accept a regimented life in a teeming world from which all wilderness and fantasy will have disappeared*” [95].

“*Just as important for maintaining the quality of human life is an environment in which it is possible to satisfy the longings for quiet, privacy, independence, initiative, and open space. These are not frills or luxuries; they constitute real biological necessities. They will be in short supply long before there are critical shortages of energy and materials to keep the human machine going and industry expanding*” [96].

In order to evaluate some of his claims—that the losses incurred by environmental deterioration, sanitization (loss of contact with biodiversity), technological overwhelm, loss of privacy, and other Paleolithic mismatches would manifest as deficits in the areas of mental health, overall quality of life, and humanness (again, defined by Dubos as altruism and empathy)—we must first determine if the manifestations are actually ongoing. In other words, are there any signs of subtle and not-so-subtle changes in emotional well-being within developed nations and those undergoing the epidemiological transition? The short answer is yes. There seems little doubt that since Dubos wrote one of his final papers, wherein he stated that the most valuable members of future technological societies would be those high in empathy (because those lacking empathy would have little understanding of how to apply rapidly developing “*scientific and technological prowess to the deepest concerns of human life*” [97]), there have been generational changes in mental health and, especially, in dimensions of personality.

In support of his contention, population studies have shown marked increases in narcissism, anxiety, depression, behavioral disorders, and declines in empathy (in particular, perspective taking and concern for others). The shifts have been most noticeable in the last 2 decades [98-104]. Research in the United States covering the span between 1995 and 2010 shows that outpatient physician visits resulting in mental disorder diagnoses per 100 population have increased significantly (up from 7.78 to 15.30 visits among children and teens and 23.23 to 28.48 visits for adults), and the use of antidepressant medication in the USA has increased by 38% since 1999 [105,106]. Scientists are increasingly taking stock of the mental health consequences of actual and perceived erosions in privacy [107,108].

While a recent meta-analysis indicates that *diagnosable* depression and anxiety disorders have remained relatively constant since 1990, international studies incorporating the General Health Questionnaire (GHQ) in assessment have, in most cases (8 of 11 studies), reported a significant increase in psychological distress over time (1977–2009 catchment in the same meta-analysis) [109]. Between 1983 and 2009, scores on the Perceived Stress Scale increased by 21% among American men and women [110]. Researchers are also just beginning to acknowledge the far-reaching and detrimental consequences of so-called sub-threshold mental disorders and steady psychological distress that would otherwise fall short of major diagnosable criteria [111-113]. One third of patients arriving in the offices of primary care physicians present with symptoms of depression, anxiety, and/or alcohol problems, yet less than 8% specifically arrive to address psychiatric symptoms [114].

While researchers and clinicians grapple with diagnostic criteria and terminology related to the definitions of broad terms such as “anxiety” and “depressive symptoms,” there seems little doubt that those sitting just outside of the debated criteria, apparently an ever-increasing percentage of the global population, are hardly in optimal mental health. The viewpoint of Dubos was along those very sight lines—less focused around an epidemic of mental disorders *per se* and more concerned with a future erosion of optimal mental health and humanism. Research certainly suggests that his future is now. Dubos argued that separation from the natural world was eroding humanness and that humanness was centrally defined by altruistic and empathic behavior.

“*However, the really human aspect of altruism is not its biological origin or its evolutionary advantages but rather the fact that humankind has now made it a virtue regardless of the practical advantages or disadvantages. Since earliest recorded history, altruism has become one of the absolute values by which humanity transcends animality*.” [115].

To be clear, as much as Dubos mentioned Paleolithic and Stone Age imprints related to modern health, he provided equal time to underscoring the fact that DNA does not determine behavioral destiny. His studies with animals paved the way for behavioral epigenetics. He proved, at least in rodents, that there are adult implications to early-life variables such as maternal grooming, social setting, stress, nutrition, and microbiota contact.

“*Through complex mechanisms that are only now being recognized, environmental stimuli determine which parts of the genetic endowment are repressed and which parts are activated. In other words, the life experiences determine the extent to which genetic endowment is converted into functional attributes. From nutrition to education, from topography of the land to religious background, countless are the attributes that contribute to shaping the body and the mind of man*” [116], [page 153].

Some 30 years later, the discovery of epigenetic changes would explain how early-life experiences can indeed be converted into functional attributes [117]. From a pessimistic perspective, the realization that environmental factors can influence neuropsychiatric risk in offspring beyond a single generation (i.e., they can be trans-generational [118,119]) does not bode well in the context of apparent generational increases in psychological distress. On the other hand, the mental health implications of epigenetic processes are cause for optimism because evidence indicates that they are modifiable [120,121].

## Natural environments

“*Urban dwellers never have the chance to see the Milky Way, or a night radiant with stars, or even a truly blue sky. They never experience the subtle fragrances peculiar to each season; they lose the exhilaration of early spring and the delightful melancholy of autumn. The loss of these experiences is more than an aesthetic affliction; it corresponds to a deprivation of needs which are essential to physical and mental sanity, because they were indelibly woven in man’s fabric during his evolutionary past*”.

René Dubos in Bailey A [122].

Dubos argued that access to and contact with natural environments was essential to the mental health of populations; therefore, an appropriate starting point is to examine in top-down fashion—i.e., through an epidemiological lens—the recent scientific assessments of the relationship between nature and mental well-being. A variety of population-level studies have provided support to the notion that natural environments are associated with health promotion in urban settings. Studies have linked the perceived degree of greenness of urban neighborhoods with individual life satisfaction and mental health among residents [123,124].

Researchers using a land-use database in the Netherlands have also discovered associations between higher levels of neighborhood green space, and both self-reported general health and a decreased risk of physician-assessed diseases. Specific to mental health, those with ≤10% green space within 1 km of the residence had a 25% greater risk of depression and a 30% greater risk of anxiety disorders vs. residents with the highest concentration of green space in proximity to the home [125,126]. A separate investigation from Denmark reported that residing more than 1 km from high green space areas was associated with a 42% increase in high levels of self-reported stress and poor scores on measurements of general health, vitality, overall mental health, and bodily pain [127].

In a USA study that included rural and urban communities, researchers compared mental health outcomes with an objectively determined vegetation index and percentage of neighborhood tree canopy coverage. It was reported that residence in areas with a high ratio of green space was associated with better mental health. Lower risk of depression was, in particular, strongly linked to neighborhood green space with each measurement—vegetation index, tree canopy, and an average of the two [128]. In a study involving urban New Zealand residents, every 1% increase in the proportion of useable or total green space in proximity to the home was associated with a 4% lower rate of anxiety/mood disorder treatment [129]. If Dubos were alive today, he would perhaps be unsurprised that decreasing the residential distance to the nearest usable green space seemed to diminish the need for clinical interventions in anxiety and depression.

Supporting this overall connection between urban green space and mental health, researchers using the British Household Panel Survey (BHPS) found that residential relocation to or from areas with more/less green space was associated with mental health. Specifically, the researchers found that (vs. premove data and controlling for income) the 594 individuals who moved to areas higher in green space had better mental health scores on each of the subsequent years. These sustained mental health improvements, observed through 3 years of study, were not realized in the years following a move to a less green area [130]. The BHPS and a land-use data set have also shown that better mental and general health is associated with residence in proximity to coastal regions—so-called blue space—an association that was observed to be independent of green space [131].

Studies involving community samples have also examined green space in relation to objective markers of stress physiology. For example, lower residential greenness is associated with higher blood pressure among 10 year-old children living in an urban Munich, Germany [132]. Studies involving socially disadvantaged urban adults in Scotland have found that salivary cortisol follows a healthy pattern in association with lower perceived stress in those residing in areas containing higher (>43% green space) [133,134]. Although better access to natural environments does seem to encourage the cardio-protective activity of recreational walking, research indicates that physical activity does not always mediate the relationship between green environments and health outcomes [135], indicating that there may be as-yet-undetermined mechanisms that explain the benefits of green space for human health.

## Epidemiology—contextual findings

Although our focus is on mental health, there are a number of additional population studies that can be considered relevant to the Dubos’ argument concerning the need for natural environments. Depressive and anxiety disorders have been found to be drivers of chronic disease states—most notably type II diabetes, cardiovascular, and gastrointestinal diseases and disorders [136-141]. Therefore, if green space is associated with mental well-being, as indicated by the research cited above, it would certainly suggest that it might also be associated with reduced mortality rates.

Indeed, a variety of international studies have linked regional forests [142], regional green space [143], walkable green space [144], urban parks [145], and overall urban green space with lowered mortality rates [146,147]. The latter study controlled for socio-economic status and found that the combination of low income and low residential-area green space was associated with cardiovascular mortality rates twice that of those living in more affluent areas. However, when low income was paired with high levels of residential-area urban green space, mortality rates for disadvantaged communities vs. affluent areas narrowed significantly [147].

Separate research on the beneficial effects of green space concentration and pregnancy outcomes (birth weight and preterm risk) and the higher risk of postpartum depression in urban vs. rural environments [148-152] indicates the far-reaching effects of natural environments. Considering that children born preterm (vs. peers delivered full term) are at significantly higher risk of mental disorders [153], a generational influence of natural environments could be possible. Also, in a sample of almost 260,000 Australians, higher percentage green space in neighborhoods (80% or more vs. 20% or less) was associated with a diminished likelihood of short sleep duration [154]. Given the links between normal sleep duration, psychological well-being, and decreased risk of obesity [155,156], these findings are of relevance to public health.

The results of the cited Australian study [154] are particularly intriguing because the link between green space and healthy sleep duration was not associated with physical activity, socioeconomic status, or current psychological distress. The daily use of natural environments might facilitate the benefits of natural light delivered to the retina at the right time of day [157]. The extent to which areas containing as much as 80% green space (as reported in [154]) is associated with *less* outdoor light at night (LAN), which in turn might influence sleep [158,159] and mental health [160], needs to be explored.

Urbanization has been linked with reduced infant sleep [161] and experimental research demonstrates long-term consequences of dim light at night in early life, on subsequent anxiety and other neuropsychiatric disorders [162]. In adults, sleep debt is known to compromise emotional empathy [163]. The degree of artificial skyglow (i.e., light pollution via scattering of light in the atmosphere) in dense urban areas can easily match and exceed than that of the brightest summer moonlight, and its ability to obscure visibility of stars and potentially place significant pressure on ecological systems is not a matter of debate [164,165]. Moreover, massive increases in man-made electromagnetic radiation to accommodate technological devices [166,167], especially in densely populated areas, may also influence the circadian systems and sleep quality [168,169].

### Dubos on light

“*Until the last century, man lived in the dark for long hours…modern man, in contrast, is exposed to bright light for 16 hours a day. In view of the fact that light rays can affect several hormonal activities, and that many, if not most, physiological functions are linked to circadian and seasonal cycles, it seems possible that this change in the ways of life will have long-range consequences for the human species*” [170].

### Dubos on radiation

“*Throughout his life and evolution, man has been exposed to a background of radiation from natural sources. This normal background, which seems to have remained fairly constant, at least in geologically recent times, must therefore be a tolerable factor of human environment…and it is unlikely that adaptive processes can occur fast enough to cope with the potential long-range dangers if man-made radiation continues to increase at its present rate*” [171].

If natural environments are working toward the promotion of public health, mental health in particular, we would expect there to be consequences when such environments are subjected to degradation. Unrelenting industrial activities, climate change, invasive species—or a combination of the lot—are examples of environmental variables that can visibly alter natural environments, sometimes in a relatively short period of time. Although the research in this area remains limited, there is evidence that environmental degradation, including changes to vegetation and tree loss at the community and regional level, is associated with increased physical illness and declining mental health, including depression and a loss of sense of place [172-177]. When researchers used three-dimensional videos to evaluated the extent to which differing urban tree density might mitigate stress induction in volunteers, the benefits were noted in a linear fashion—greater density of urban street tree canopy was associated with more effective self-reported stress recovery. As the researchers point out, it appears that every tree matters [178].

The epidemiological research related to natural environments does have some shortcomings, not the least of which includes a common reliance upon cross-sectional design and the use of self-reports. However, the population research does not stand alone. It is bolstered by a variety of *in vivo* and experimental studies which point in the direction of Dubos—i.e., an evolutionary need for natural environments, one that stands apart independently of other variables, the awareness of which could be masked in the midst of shifting priorities.

In his words concerning the future of the human masking of that need—“*In fact, he may soon forget that some of his most exhilarating experiences have come from direct contact with freshness, brilliance, and rich variety of unspoiled natural phenomenon. Unfortunately, perhaps, starless skies and joyless sceneries are not incompatible with the maintenance of life, or even with physical health. The only measure of their loss may be a progressive decadence in the quality and sanity of the human condition*” [179].

## Natural environments—*in vivo* research

Increasingly, researchers are engaging in studies that compare activities (most often walking) in natural environments vs. the same activity conducted in the built environment. In an effort to advance the quality of the research, investigators are beginning to employ pre/post-objective measurements of physiology and utilize validated instruments of neuropsychological relevance. For example, researchers induce neurocognitive fatigue with mentally challenging tasks designed to place demands on sustained attention. Immediately following this intense cognitive effort, the subjects take a walk (varying times, typically 30–60 min) in a vegetation-rich park or on city streets and at the conclusion of the walk, neuropsychological tests are repeated. Using this general design, researchers have reported more significant cognitive benefits post-nature walk in healthy adults [180,181], children with deficits in attention [182], and adults with depression [183].

Some of the research indicates that this so-called cognitive restoration was occurring without changes in emotional state *per se*, suggesting that the cognitive benefits are not merely the result of acute positive mental outlook [183]. Separate research, involving 56 adults engaged in a wilderness hiking excursion, found that 4 days into the hike the scores on the Random Associates Test (used for creative thinking and insight problem solving) were 50% higher vs. pre-hike scores [184]. Research involving 51 university students has also showed that when subjects are randomly queried (via pager at various times between 10a and 10p) about current activities and vitality, even when the presence of others, physical activity and outdoor environments in general were controlled for, the presence of natural elements was a mediator of subjective vitality [185].

Added to the weight of such studies is a large volume of research under the umbrella term of *shinrin-yoku* (translates from Japanese as “forest bathing” or “taking in the forest”) and related descriptive of forest therapy. Reviews and original research on the subject through 2010 captured nine studies involving field work evaluating forest walking or comfortably seated viewing/contemplation of the forest (vs. urban built) environment. These studies have reported, perhaps unsurprisingly, that the forest experience improved subjective mood state. However, the mood changes have been corroborated with physiological findings—e.g., lower cortisol levels, reduced sympathetic tone, improved natural killer cell count/activity, lowered pulse rate and blood pressure, and improved heart rate variability [186,187]. Since the publication of these reviews, additional studies, including those from China and Korea, have documented similar improvements in subjective mood and end points such as stress hormones, oxidative stress, systemic inflammation, and blood pressure [188-196].

## Natural environments—objective markers

The argument that natural environments may be a deeply rooted means to buffer contemporary stress is strengthened by the results of objective neurophysiology and imaging studies. For example, electroencephalograph (EEG) has reported higher alpha wave activity, suggesting a state of relaxed wakefulness and lowered anxiety, when viewing scenes of natural environments [197-199]. Changes in real-time EEG measurements during a 25-min walk (participants walked from an urban shopping district through a vegetation-rich urban park) were associated with emotional parameters indicating less frustration, higher engagement, and a more meditative state while transitioning through green space [200]. Japanese researchers have used near-infrared time-resolved spectroscopy (NITRS) to measure oxygen use in the brain via the reflection of near-infrared light from red blood cells. In separate reports, they have shown that viewing an outdoor forest setting (vs. urban control), actual plant foliage (vs. projected images), and a three-dimensional plant image (vs. two-dimensional), changed cerebral oxygen use in ways that might substantiate subjective reports of mental focus and/or relaxation [201-203].

Several functional magnetic resonance imaging (fMRI) studies have demonstrated divergent brain activation patterns during the presentation of urban built vs. natural environment scenery [204-207]. With relative consistency in these studies, scenes of natural environments increased activity in regions associated with positive affect, emotional stability, altruism, empathy, and depth of love. The urban scenes, in contrast, increased activity in the amygdala, an area well-documented to be a hub in arousal, risk, and threat detection. Interestingly, it has been reported that urban adults (or those who grew up in an urban vs. rural environment) have increased activity in the amygdala while performing challenging tasks and researcher-induced perceived social stress [208].

Separate fMRI work has revealed that images of blue sky with some green vegetation can activate the same areas of the brain that are provoked by positive (vs. neutral or negative) images. In addition, the sky images activated unique areas associated with expanse of space, circadian rhythms, and dreaming [209]. The extent to which environmental colors (e.g., blue and green) mediate brain activation in areas associated with emotional response is an active area of investigation [210].

Researchers have also used scenes of natural environments to determine if they may influence markers of stress physiology. For example, in the immediate period following a laboratory stressor, markers of stress physiology (electromyography, skin conductance, and/or pulse transit time show a more rapid return to baseline after viewing scenes of nature vs. urban environments deficient in vegetation [211]. Spending time in nature, activities associated with gardening, or simply viewing scenes of nature, has been associated with favorable responses as measured by autonomic control and heart rate variability [212-216]. For example, viewing scenes of nature vs. an urban built environment for 10 min *prior* to a mental stressor results in enhanced heart rate variability and more dominant parasympathetic activity [216]. These studies are not confined to the visual system alone; there is also research indicating similar physiological and mood effects using auditory (sounds of nature), olfactory (volatile chemicals released from leaves), and even tactile (touch of real leaf vs. synthetic leaf made of resin) stimuli [217-222].

## Natural environments—cognition, altruism, and discounting

Several studies have found inverse relationships between childhood play experiences in green/blue spaces and hyperactivity and/or inattention [223-226]. One of those studies also found that beach attendance among children aged 7–11 is associated with pro-social behavior [227]. A growing body of research also suggests that natural environments may be an effective means to mitigate some of the consequences of the cognitive load characteristic of modern urban environments [228,229].

The results of laboratory experiments certainly validate the notion that viewing scenes of nature (vs. urban built scenes) can lead to more rapid recovery of cognitive performance (executive attention, memory recall, target identification, reaction time, logical reasoning, and anagram task performance) in the period following challenging activities known to induce mental fatigue [230-234]. Using a similar *modus operandi* of induced cognitive fatigue prior to field-setting walks, researchers find performance benefits after walking in nature vs. the urban built environment [181,183]. There are also indications that proximity of academic settings in relations to natural environments, and classroom views to natural environments, may be associated with positive academic performance [235-237]. Similar findings concerning benefits of window views to nature (or the presence of indoor potted plants) have been reported where the end point was workplace performance [238-240].

If natural environments can improve positive emotions and limit cognitive fatigue, they may also help with future discounting and the erosion of helping behavior associated with cognitive load. Volumes of research show that humans discount the value of future rewards, instead prioritizing smaller immediate rewards. Greater discounting of the future in favor of a small reward has been associated with impulsivity, depression, obesity, and a host of detrimental lifestyle habits [241-243]. The overall cognitive load within an urban environment, perceived competition for resources, and even physical aspects of the built environment may magnify delay discounting. Indeed, researchers have found that viewing scenes of nature or being in urban natural environments (vs. control scenes of, and actually being in, the urban built environment) is associated with significant reductions in discounting the future. Nature scenes or being in nature minimizes impulsivity and enhances the value assigned to future reward [244,245].

Moving toward altruism, researchers have found that exposure to images of natural environments (vs. built environment) is associated with increased intrinsic aspirations (e.g., depth of meaning within relationships, personal growth, and community value) and devalued extrinsic aspirations (e.g., accumulation of wealth, personal image, status, and fame). Similar results were found when (without participant knowledge) four plants were either present or absent in the laboratory)—higher value was placed on intrinsic aspirations when plants were in the room. The presence of plants was also associated with increased generosity in an economic decision-making task [246].

A series of field experiments demonstrated increased helping behavior (returning a “lost” glove) after random subjects had walked through a natural environment within a town center [247]. The helping behavior appeared to be mediated by positive mood induction via very brief periods (approximately 1 min) spent in the natural environment. In addition, the induction of awe by nature scenes has also been shown to direct attention away from the self and toward the environment, increasing feelings of connectivity to others, and enhancing the desire to spend time in nature or take on creative pursuits [248].

Of relevance to the hurried urban existence, where the *perception* of lack of time encourages unhealthy lifestyle habits, it is noteworthy that the induction of awe by nature scenes has been shown to increase the perception of time availability. In turn, this translates into decreased impatience and increased willingness to volunteer. When awe is induced, there is also a preference for experiential rewards (e.g., an event) vs. rote material goods (e.g., a wristwatch) of equal value [249].

This research certainly supports the humanistic view of Dubos. His assertions that ongoing technological and cultural changes would mask awareness of the potential of health via natural environments—i.e., that it would not be consciously missed—have been indirectly evaluated by noted Canadian researcher Elizabeth Nisbet and colleagues. Although there may be no historical data with which to compare, Nisbet has consistently found that contemporary Westernized adults undervalue the psychological value of nature [250]. For example, she has determined that prior to engaging in 17-min walk in urban green space (vs. indoor environments), adults consistently undervalue the subsequent actualized improvement in positive affect. Importantly, the researchers found that walking in the outdoors facilitated a sense of nature relatedness [251]. Fostering a connection to nature or awareness of its benefits provides an optimistic perspective. Higher scores on validated scales can capture an individual’s “nature relatedness”, “connectedness with nature”, and “nature connectivity”; higher scores are significantly correlated with lower anxiety and anger, a more positive mental outlook, greater well-being, and overall vitality [252-256].

The link between nature connectivity and personal well-being is found in student and community samples of adults with broad-ranging ages, occupations, and health status [252-254]. Researchers are untangling the psychological mediators that appear to strengthen the connections between nature, helping behavior, cognitive function, and personal well-being; they include the degree of awareness/mindfulness, meaning in life, perceptions of beauty, and positive affect [257-261]. We wonder what the epigenetic implications of such mediators might be.

Critically, higher scores on nature relatedness are also associated with pro-environmental attitudes—greater concern for all living things (see Biophilia and Biophilism section below), the broad community and future generations [250,254]. Higher scores on the nature relatedness scales are also positively and significantly associated with empathy [262]. Short-term induction of nature connectivity, should it be consistent over time, may offer a means to improve some aspects of personal health and positive attitudes toward the environment, although these long-term benefits from isolated short-term nature experiences in urban settings remain speculative [263].

It should be pointed out that the benefits of nature interventions on psychological well-being do not seem to be predicated on an individual’s baseline nature relatedness, and these benefits, in turn, appear to motivate the individual to maintain nature-based experiences [264]. Therefore, it seems reasonable to conclude that *opportunity* is a prerequisite to develop a connection to nature. Over time, this may influence attitudes—for example, it is noteworthy that the perceived potential of a natural environment to produce cognitive restoration may be linked to pro-environmental attitudes of the users [265].

## Natural environments—benefits of biodiversity

As we transition our discussions toward other potential evolutionary mismatches (or paleo-deficits) in Part II, we conclude Part I with the topic of biodiversity. Dubos had much to say concerning the loss of biodiversity and forewarned that discussions of biodiversity loss should not be privileged by select species that are emotionally favored by humans. Even the lowly microbe was as relevant as the Giant Sequoia in his biodiversity discussions:

“*man himself has emerged from a line descent that began with microbial life, a line common to all plant and animal species*…[he] *is dependent not only on other human beings and on the physical world but also on other creatures*—*animals, plants, microbes*—*that have evolved together with him. Man will ultimately destroy himself if he thoughtlessly eliminates the organisms that constitute essential links in the complex and delicate web of life of which he is a part*” [266].

Of course, rapid urbanization is a significant driver of global biodiversity loss [267]. In turn, given the critical role of biodiversity in global health [268-270], the impacts of its loss may also be stealth-like, tallied only through the aggregate of public health statistics. Moreover, the psychological *benefits* of local biodiversity may also be without conscious realization. For example, research has shown that as it relates to human health and well-being, the benefits of urban green spaces appear to be related to the extent of their overall biodiversity [271-273].

Remarkably, when humans hear birdsongs in the background of an experimental setting in which they are tasked with rating urban environments, their appreciation for scenes of urban landscapes is increased along a gradient coincident with the diversity of audible birdsongs [274]. We have much to learn about the ways in which biodiversity has shaped us. However, it seems apparent that, as Dubos likely would have argued, we cannot make up for these potential losses with a birdsong-generating machine from a high-end gadget outlet.

## Other paleo-deficits

Emerging studies certainly support Dubos in his contention that revisiting certain “Paleolithic experiences,” at least the more pleasant ones (vs. those that might have helped develop our efficient fight or flight response), could have a positive influence on metropolitan quality of life. However, the modern urban environment may be *missing* more than merely green/blue space, natural sounds, and light at the right time. The ability of green spaces to facilitate social contacts is an important consideration [275]. Children who reside in close proximity to urban green spaces and forests are significantly *less* likely to engage in excessive screen time [276]; therefore, the dialogue on natural environments cannot take place in isolation. As we outline in Part II, there are other interrelated discussions that often escape discourse in the context of natural environments yet may be no less relevant with regard to what is *missing* in the modern landscape vs. our ancestral past—diversity of microbes and traditional dietary patterns.

### Biophilia and biophilism—some history

It is commonly stated that biophilia (Greek—*bios* (life), *philia* (love)) is a 20th century neologism that was “coined” in the 1980s. However, the term biophilia appeared in many 19th century medical and psychology dictionaries as meaning not only love of life but also *the instinct for self-preservation common to man and the lower animals* [277]. It was Charles J. Adams, a religious scholar and author, who popularized the term biophilism in the late 19th and early 20th century.

In 1895, Adams formed a “Bureau of Biophilism”, which included the well-known naturalist John Burroughs, poets Henry Abbey and Eugene Field, dog expert Eugene Glass, et al. [278]. Writing in professional as well as lay periodicals such as *Forest and Stream* and *Dog Fancier* [279,280], Adams argued that biophilism was not merely the love of one’s own life, it was also the love of non-human life, even that of lower animals. “*Biophilism means the love of life. The love of what sort of life*”? Adams queried in a 1907 article [281], answering his own question by stating that “*out of self love one should come into…love of humanity, out of love of humanity into love of all sentient things. When he has so far evolved, he is a biophilist. As there may be born a poet, so there may be born a biophilist*”. However, Adams argued that although birth can provide the ingredients for biophilism, it could only be realized if the innate powers, as he called them, were “*more than drawn out. They must be developed, trained*” through experience.

References to biophilia and biophilism waned until the mid-20th century when psychologist Eric Fromm provided some renewed enthusiasm for the term as it pertains to mental state. Although Fromm made little reference to nature or the environment *per se*, others did so for him. For example, in 1969 scholar Michael McGrath interpreted Fromm’s biophilia [282] in a way that might be captured by contemporary Nature Relatedness Scales—“*A simple form of biophilous behavior is that of plants leaning toward the source of sunlight…biophilia confirms and promotes life. The biophilous person produces rather than destroys, creates rather than hoards. He is more interested in living things, such as nature and other people, than he is* ‘*dead*’ *things such as sports cars and spaceships*”.

Biophilia, as in the love of all life on Earth, was subsequently positioned in the early 1970s as a potential benefit to environmental conservation [283]. In 1979, biologist Edward O. Wilson argued the following in *The New York Times*—“*Our deepest needs stem from ancient and still poorly understood biological adaptations. Among them is biophilia: the rich, natural pleasure that comes from being surrounded by living organisms, not just other human beings but a diversity of plants and animals that live in gardens and woodlots, in zoos, around the home and in the wilderness*” [284]. Dubos, to our knowledge, never used the term biophilia, preferring instead a “*biological joie de vivre*” that could be obtained through solidarity with other forms of life.

Said Dubos—“*…the essential factors of biological joie de vivre exist in every human being because they are inscribed in the genetic code. In fact, this aspect of life has probably not changed significantly since the Stone Age…the purely biological enjoyment of life can, in addition, evolve into a more subtle experience of universal fellowship with all other human beings and even with other forms of life…ever since the Stone Age, and in all parts of the world, human beings have expressed their awareness of solidarity with other forms of life*….” [63].
